# Effect of Oral Bromelain on Wound Healing, Pain, and Bleeding at Donor Site Following Free Gingival Grafting: A Clinical Trial

**Published:** 2018-09

**Authors:** Sara Soheilifar, Mohsen Bidgoli, Amirarsalan Hooshyarfard, Armaghan Shahbazi, Farshid Vahdatinia, Fahime Khoshkhooie

**Affiliations:** 1 Assistant Professor, Department of Periodontics, School of Dental Medicine, Hamadan University of Medical Sciences, Hamadan, Iran; 2 Postgraduate Student, Department of Periodontics, School of Dental Medicine, Hamadan University of Medical Sciences, Hamadan, Iran; 3 Assistant Professor, Department of Prosthodontics, School of Dental Medicine, Hamadan University of Medical Sciences, Hamadan, Iran; 4 Dental Surgeon, Dental Research Center, School of Dental Medicine, Hamadan University of Medical Sciences, Hamadan, Iran; 5 Private Dentist, Hamadan, Iran

**Keywords:** Bromelain, Wound Healing, Transplant Donor Site, Operative Surgical Procedure

## Abstract

**Objectives:**

Considering the optimal efficacy of bromelain for pain relief and wound healing, this study aimed to assess the effect of bromelain on wound healing, pain, and bleeding at the donor site following free gingival grafting (FGG).

**Materials and Methods:**

This randomized, controlled double-blind clinical trial was performed on 26 patients with gingival recession. The patients were randomly divided into two groups of bromelain and placebo (n=13). Treatment was started on the day of surgery and was continued for 10 days. Pain, bleeding, and epithelialization at the donor site were the variables evaluated in this study using a questionnaire. The level of pain was determined using a visual analog scale (VAS) considering the number of analgesic tablets taken within 7 days postoperatively. Bleeding was determined according to the patient’s report, and epithelization was assessed by applying 3% hydrogen peroxide (H_2_O_2_) to the donor site. The donor site epithelialization was assessed at 7 and 10 days after surgery.

**Results:**

Bromelain caused a significant reduction in pain at the donor site (2.605±0.509) compared to the placebo (4.885±0.519; P<0.05). The number of donor sites with complete epithelialization was higher in the bromelain group compared to the placebo, but this difference was not statistically significant (P>0.05). The two groups were the same regarding postoperative bleeding (P>0.05).

**Conclusions:**

The results showed that oral bromelain (500 mg/day) can be effective in the reduction of pain at the donor site after FGG and may also enhance wound healing. Oral bromelain does not increase the risk of postoperative bleeding.

## INTRODUCTION

Root coverage for treatment of gingival recession is performed to improve esthetics, to enhance plaque removal around the gingival margin, and to resolve inflammation around restored teeth [1]. Several techniques are used for this purpose, and free gingival grafting (FGG) is among the most common approaches [1].

Pain and bleeding at the donor site are among the most important complications of FGG [1]. In this technique, the donor site is sutured after graft harvesting and is protected with a periodontal dressing for one week. In some cases, the dressing needs to be maintained for a longer period of time [1]. Use of a palatal stent as a plastic coverage has also been suggested for some patients since it compresses the area and decreases bleeding. Moreover, it protects the palate from the trauma of eating [2]. Nonsteroidal anti-inflammatory drugs (NSAIDs) are often administered for pain control. However, the healing period is often long, and no definitive method has been suggested to decrease donor site complications in these patients [2,3].

According to animal and human studies, bromelain is an edible herbal product with analgesic and anti-inflammatory properties. Bromelain is a proteolytic enzyme derived from the stem of the pineapple plant as a complex mixture of different thiol-endopeptidases, phosphatases, glucosidases, peroxidases, cellulases, glycoproteins, and carbohydrates [3–5]. Bromelain directly affects pain mediators such as bradykinin, decreases swelling and bruise, and shortens the healing period following trauma and surgical procedures [6–9]. Also, Bromelain reduces plasma kininogen, thus inhibiting the production of kinin, which is known as an agent that induces inflammation, pain, and swelling [3]. Thus, researchers are evaluating its efficacy to enhance healing after plastic surgery [10]. Evidence shows that bromelain is more effective if administered orally [10]. Bromelain is well tolerated by the gastrointestinal (GI) system, and animal studies have shown that it has no toxic effect up to 10 g/kg body weight [11,12].

Considering the positive effects of bromelain mentioned earlier, this study aimed to assess the effect of bromelain intake on pain, bleeding, and wound healing at the donor site after FGG.

## MATERIALS AND METHODS

This randomized, controlled double-blind clinical trial is registered at the Iranian Registry of Clinical Trials (IRCT201502089002N5) and has been approved by the Ethics Committee of Hamadan University of Medical Sciences (P/16/35/9/4782). Twenty-six patients requiring FGG for treatment of gingival recession in the anterior mandible were enrolled according to our eligibility criteria. The inclusion criteria comprised the presence of Miller’s Class I, II, or III gingival recession in one of the mandibular incisors, systemic health, no intake of anticoagulants such as warfarin, no use of antibiotics or corticosteroids in the past two months, and no cigarette smoking. The exclusion criteria comprised not showing up for any of the follow-up sessions, medication intake other than those prescribed for this study, poor oral hygiene during the healing period, and not clearly disclosing the number of analgesics taken. After patient selection, scaling and prophylaxis were performed if required, and the patients received oral hygiene instructions. The patients were then randomly assigned to the control or case groups using a table of random numbers. FGG was performed by a surgeon one month after scaling and oral hygiene instruction. The standard surgical approach was adopted for the procedure, and root planing and recipient site preparations were performed. Next, a partial thickness graft was harvested from the palate. The respective flap was trapezoidal with 10 mm height and 7 mm length of the small base (this size would fit most of the gingival recession sites in mandibular incisors). The donor site was rinsed with saline and sutured such that the distance between sutures was not more than 2 mm. The harvested graft was then trimmed to the suitable thickness, and its underlying fat was eliminated in order to prevent interference with the process of vascularization. Next, the graft was implanted at the recipient site and fixed by sutures. Both the donor and recipient sites were protected using a periodontal dressing. In case of loss of dressing, the patients were requested to refer for a new dressing. After the surgical procedure, antibiotics (500 mg amoxicillin, three times a day) and analgesics (400 mg Gelofen, every 8 hours) were prescribed for patients in both groups. Also, 0.2% chlorhexidine mouthwash was prescribed for one week. The patients were instructed to perform regular tooth brushing, except for the graft site. Also, they were requested to write down the number of analgesics taken and report it on the day of suture removal.

### Administration of bromelain:

For the case group, bromelain capsule (500 mg; Tasnim Pharmaceutical Co., Tehran, Iran) was prescribed once a day for 10 days, starting from the day of surgery [13]. A placebo with the same packaging as bromelain (produced at the School of Pharmacy, Hamadan University of Medical Sciences, Hamadan, Iran) was administered in the control group with the same dosage and frequency of intake.

### Preparation of placebo:

The placebo powder included 90% microcrystalline cellulose (Avicel^®^) + 5% corn starch + 2% magnesium stearate + 3% FD&C yellow dye (to mimic the color of bromelain capsule).

The drugs were administered among the patients by a third examiner. Neither the surgeon nor the patients were informed about the type of medication. Both medications had the same packaging. At 7 days postoperatively, patients in the two groups were assessed and compared in terms of pain, bleeding, and epithelialization at the donor site. Data were collected and recorded in a questionnaire by the examining surgeon. The level of pain was assessed using a visual analog scale (VAS) from 0 to 10 (zero indicated no pain, while 10 indicated the worst pain imaginable) according to the number of analgesics taken.

To assess the epithelialization, the surgeon dried the donor site with a gauze and applied 3% hydrogen peroxide (H_2_O_2_) using a microbrush [14]. In case of complete epithelialization, the H_2_O_2_ would not penetrate into the connective tissue and would not subsequently release oxygen; thus, no bubbles would be seen. Otherwise, epithelialization was considered to be incomplete. This was repeated on day 10 postoperatively. The same assessment was also done on day 14 postoperatively. However, since epithelialization had already completed in all the patients at this time, day 10 postoperatively was chosen for the comparison of epithelialization between the two groups.

### Data analysis:

Data were analyzed using SPSS software (Version 20; SPSS Inc., Chicago, IL, USA) via descriptive (mean, standard deviation (SD), and frequency) and inferential statistics (T-test, Chi-square test, and Fisher’s exact test for bleeding, Poisson regression for the number of analgesics, and logistic regression) at 0.05 level of significance. The study power was 90% in all tests.

## RESULTS

A total of 26 patients were evaluated in this study, out of which, 38% were males and 62% were females in the control group, and 31% were males and 69% were females in the case group. The mean (±SD) age of the patients in the control and case groups was 27.83±8.674 and 28.23±7.991 years, respectively, and the mean (±SD) number of analgesics taken was 4.38±4.34. Overall, 18% of the patients showed bleeding, and the mean (±SD) score of pain was 3.84±2.29. Of all, 53.8% showed complete epithelialization on day 7 postoperatively; this value was 80.8% on day 10. As shown in Table 1, the mean (±SD) number of analgesics taken was 5.46±5.34 in the control group and 3.31±2.86 in the case group. Since the number of analgesics taken in the two groups showed the Poisson distribution, Poisson’s regression was used to compare the two groups in terms of the mean number of analgesics. The results showed that, averagely, the patients in the case group took half the number of analgesics taken by the controls (e^− 0.501^), and this difference was statistically significant (P=0.009; Table 1).

**Table 1.** Comparison of the number of analgesics taken by the patients in the case and control groups

** Group **	** N **	** Min **	** Max **	** Mean **	** SD **	P-value ^*^
** Control **	13	0	20	5.46	5.34	0.009
** Case **	13	0	10	3.31	2.86	
** Total **	26	0	20	4.38	4.34	

* Poisson regression test, N=Number of samples, SD=Standard Deviation

**Fig. 1:**
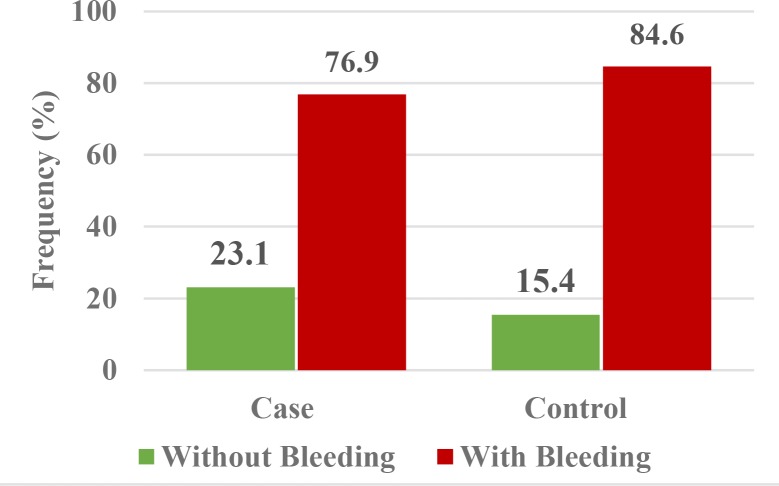
Frequency distribution of bleeding in the case and control groups

Since none of the patients in the control group had severe bleeding requiring hospital admission, we combined patients with moderate and mild bleeding to compare patients in two groups of with and without bleeding. Comparison of the control and case groups regarding bleeding showed that the frequency of patients with/without bleeding was not significantly different between the case and control groups (Fig. 1). According to Fisher’s exact test, the two groups were not significantly different in terms of bleeding (P>0.05; Table 2).

**Table 2.** Comparison of the case and control groups in terms of bleeding

** Group **	** N (%) **	** Without bleeding (%) **	** With bleeding (%) **	P-value ^*^
** Control **	13 (100)	11 (84.6)	2 (15.4)	>0.05
** Case **	13 (100)	10 (76.9)	3 (23.1)	
** Total **	26 (100)	21 (80.8)	5 (19.2)	

* Fisher’s exact test, N=Number of samples

The results showed that the intervention had no effect on bleeding in general, irrespective of other variables. A logistic regression model was used to assess the effect of the intervention on bleeding in patients that were matched in terms of other variables. The results showed that the patients matched in terms of gender, age, and the number of analgesics taken in the two groups of case and control were not significantly different in terms of the percentage of cases without bleeding. In other words, the odds of no bleeding in a control patient was 1.5 times (e^0.276^) the value in a sex- and age-matched patient in the case group; the difference in this respect was not significant (P=0.799).

The mean (±SD) pain score was 4.88±0.51 in the control group and 2.60±0.50 in the case group. First, the normal distribution of data and equality of variances were assessed in the two groups. Levene’s test showed homogeneity of variances for pain score in the two groups (F=0.712, P=0.556). Considering the fit of the variance model and by controlling the effect of age, the number of analgesics taken, and gender, the effect of group on pain score was significant according to Fisher’s exact test (P=0.006). Averagely, the mean pain score in the case group was lower than that in the control group by 2.279 units.

A lower percentage of patients in the control group, compared to the case group, showed complete epithelialization on day 7; however, Chi-square test showed no significant difference in this respect between the two groups (P=0.431). The results showed that the intervention had no significant effect on the completion of epithelialization on day 7, irrespective of other variables. A logistic regression model was applied to assess the effect of the intervention on epithelialization on day 7 after controlling other variables.

**Fig. 2:**
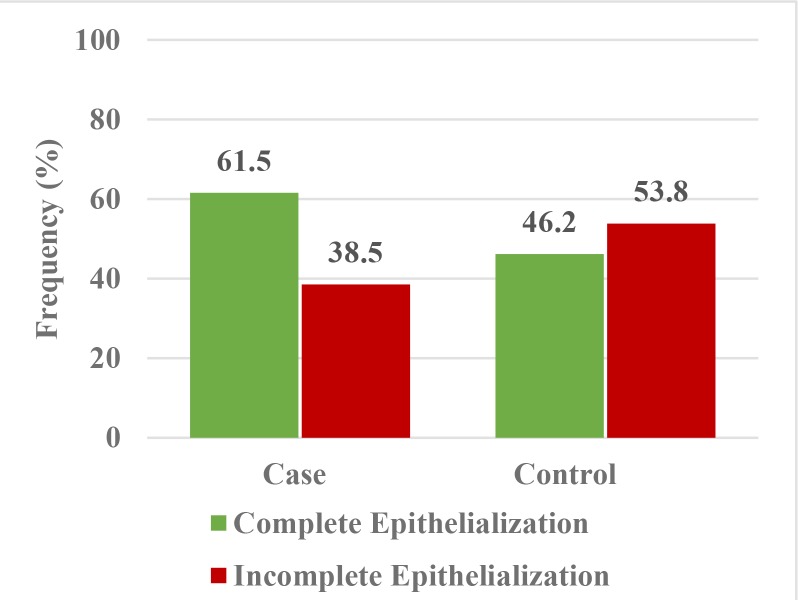
Frequency distribution of complete/incomplete epithelialization in the case and control groups at 7 days postoperatively

The results showed no significant difference in epithelialization on day 7 between the patients matched in terms of age, gender, and the number of analgesics taken in the two groups (P=0.952). In other words, the odds of complete epithelialization on day 7 in a patient in the case group were almost equal to that in an age- and sex-matched patient in the control group taking an equal number of analgesics (e^− 0.055^; Fig. 2).

Of all, 76.9% of the control and 84.6% of the case group subjects showed complete epithelialization at the donor site on day 7 after surgery. According to Chi-square test, the difference in this respect was not significant between the two groups (P=0.619).

**Fig. 3:**
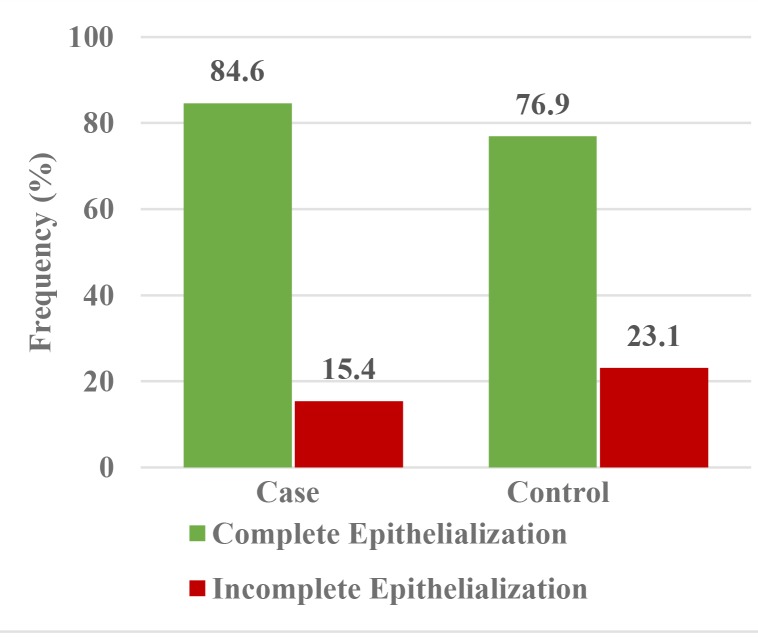
Frequency distribution of complete/incomplete epithelialization in the case and control groups at 10 days postoperatively

The results showed that the intervention had no significant effect on the completion of epithelialization at 10 days postoperatively, irrespective of other variables. A logistic regression model was applied to assess the effect of the intervention on the completion of epithelialization at 10 days postoperatively after controlling the confounding variables. The results showed no significant difference in epithelialization on day 10 between the patients matched in terms of age, gender, and the number of analgesics taken in the two groups (P=0.694). In other words, the odds of incomplete epithelialization on day 10 in a patient in the case group were almost half the value in an age- and sex-matched patient in the control group taking an equal number of analgesics (e^−0.578^); this difference was not significant between the two groups (P=0.694; Fig. 3).

## DISCUSSION

Considering the optimal efficacy of bromelain for pain reduction and enhancement of wound healing documented in previous studies [15,16], the present study aimed to assess the effect of bromelain intake on pain, bleeding, and wound healing at the donor site following FGG. The most important finding of this study was the significant analgesic effect of bromelain compared to the placebo, which was in line with the results of previous studies [15,16] although they evaluated the efficacy of bromelain for pain following other types of surgical procedures.

Majid and Al-Mashhadani [15] evaluated the effect of oral bromelain administration (1000 mg/day) on postoperative pain in patients who underwent surgical extraction of impacted mandibular third molars. They reported that bromelain significantly decreased pain compared to the placebo [15]. Inchingolo et al [16] evaluated the efficacy of a 240-mg daily dose of bromelain for controlling the edema following third molar extraction surgery and concluded that the efficacy of bromelain is similar to that of ketoprofen in the reduction of pain and edema. However, our findings were different from those reported by de la Barrera-Núñez et al [17] who assessed the efficacy of bromelain with a daily dose of 150 mg for pain relief after third molar extraction surgery and showed that the bromelain and placebo groups were the same in terms of the pain score.

All the above-mentioned studies evaluated the efficacy of bromelain for pain control following third molar extraction surgery. Considering the fact that the trauma applied to tissues and consequently the post-surgical pain after third molar extraction surgery is greater than that after graft surgery, the effective dosage for pain control in our study cannot be compared to that in previous studies.

In the study by Majid and Al-Mashhadani [15], the administered dosage of bromelain was twice the dosage in our study. However, we obtained the same result in terms of pain control. In the study by Inchingolo et al [16], the administered dosage of bromelain was 240 mg/day, and no analgesic was given to the patients in the bromelain group. However, in our study, the dosage was 500 mg/day, and the patients in the bromelain group were given analgesics as well. Nevertheless, we took into account the effect of analgesic intake on pain score as well. Both studies showed a significant reduction of pain following the use of bromelain [16].

Thus, considering the different dosages of bromelain administered in different studies, the ineffectiveness of the drug in the study by de la Barrera-Núñez et al [17] may be due to the low dose of the drug. These differences in the results of studies can be due to inadequate knowledge about the effective dosage of the drug for the reduction of postoperative pain and discomfort [17].

Studies that evaluated the effect of bromelain on isolated human platelets in vitro showed that bromelain prevents thrombin-induced platelet aggregation [18–20]; thus, it has the potential to increase the risk of postoperative bleeding. However, no hemorrhagic disorder has been reported following the use of bromelain in humans [21]. In our study, bromelain did not increase the frequency or the severity of postoperative bleeding in the case group compared to the placebo group. Only one patient in the bromelain group reported severe bleeding, which occurred during the first night after the surgical procedure and following the use of only one dose of the drug. The bleeding stopped spontaneously and did not recur during the next days; therefore, it cannot be related to bromelain intake. However, care must be taken not to prescribe bromelain for patients with coagulative disorders or for those using anticoagulants. In suspected patients, coagulation tests should be ordered prior to the prescription of bromelain.

Moreover, the number of patients in whom donor site epithelization had completed at 7 days postoperatively was higher in the case group compared to the control group; however, this difference was not statistically significant. The difference in this respect was not significant between the two groups at 10 days postoperatively either. In other words, bromelain had a positive, but insignificant, effect on wound healing at the donor site.

Maluegha et al [22] showed that the administration of the highest dosage of bromelain decreases the level of vascular endothelial growth factor (VEGF) which is also a survival factor for endothelial cells as it prevents the endothelial apoptosis induced by serum starvation. Several reports have shown that VEGF increases re-epithelialization at wounded sites [22–25].

Considering the limitations with regard to patient cooperation, the method of assessment of epithelialization in our study was based on complete epithelization at the donor site. This method did not allow for the comparison of the degree of epithelization at different time points. In fact, partial epithelization in one patient could have been greater than that in another patient. Nevertheless, an accurate comparison was not feasible. Moreover, the same method of assessment might have yielded a significant difference in a larger sample size.

Comparison of the effect of the administration of different dosages of bromelain assessed by previous studies can help in finding the most efficacious dosage and frequency of administration for pain control. However, we recommend a split-mouth clinical trial for a better comparison of results and more reliable outcomes.

## CONCLUSION

Bromelain administration (500 mg/day) can effectively decrease pain in patients after FGG. Bromelain has no negative effect on postoperative bleeding and may enhance wound healing.
